# *Lactobacillus rhamnosus* PB01 Oral Supplementation Alleviates Metabolic and Reproductive Dysfunctions in Type 1 Diabetic Male Rats

**DOI:** 10.1002/mnfr.70157

**Published:** 2025-07-04

**Authors:** Sihan Liu, Hiva Alipour, Malwina Ulanowska, Vladimir Zachar, Fereshteh Dardmeh

**Affiliations:** ^1^ Regenerative Medicine, Department of Health Science and Technology Aalborg University Gistrup Denmark

**Keywords:** diabetes mellitus, male infertility, metabolic dysfunction, probiotics, reproductive hormone

## Abstract

This study investigated the potential therapeutic effects of *Lactobacillus rhamnosus* PB01 supplementation on metabolic and reproductive dysfunctions in Type 1 diabetes mellitus (T1DM) rat model. Diabetic rats treated with *L. rhamnosus* PB01 (1 × 10⁹ CFU/day) showed significant improvements in metabolic and reproductive outcomes. Specifically, diabetes‐induced weight loss was mitigated by approximately 13%, and the percentage of hyperactivated sperm in the treatment group was significantly 2.5 times higher than that of the diabetic controls. Additionally, testosterone levels were significantly elevated by 27%, further supporting the positive effects on reproductive health. The probiotic supplementation also led to improvements in insulin sensitivity, as evidenced by a 34.12% reduction in homeostasis model assessment index of insulin resistance (HOMA‐IR). *L. rhamnosus* PB01 (DSM 14870) demonstrated potential as an adjunct therapy for managing T1DM‐associated complications in the rat model, by improving insulin sensitivity, supporting weight management, and partially restoring diabetes‐related fertility impairments, likely through modulation of hormonal pathways. However, further studies are needed to translate the results to humans and refine optimal dosing and supplementation duration for sustained benefits.

## Graphical abstract

Type 1 diabetes mellitus is known to impair reproductive function in males of reproductive age. This study investigated the effects of a 7‐week course of oral supplementation with the probiotic *Lactobacillus rhamnosus* PB01 on metabolic and fertility parameters in diabetic male rats. Supplementation resulted in notable improvements in body weight, insulin sensitivity, testosterone levels, and sperm quality. These findings suggest that probiotics may help counteract the adverse effects of diabetes on male fertility.

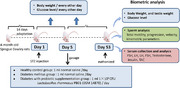

**Abbreviations**
ALH: amplitude of lateral head displacement
BCF: beat‐cross‐frequency
CASA: computer‐aided sperm analysis
DM: diabetes mellitus
DM+PB: diabetes with probiotic supplementation
FSH: follicle‐stimulating hormone
HA: hyperactivated sperm
HC: healthy control
HOMA‐IR: homeostasis model assessment index of insulin resistance
LH: luteinizing hormone
LIN: linearity
*L. rhamnosus*
: *Lactobacillus rhamnosus*
SCA: sperm class analyzer
STR: straightness
T1DM: type 1 diabetes mellitus
TAC: total antioxidant capacity
VAP: average path velocity
VCL: curvilinear velocity
VSL: straight‐line velocity
WOB: Wobble

## 1 Introduction

Diabetes mellitus (DM) is a critical global health concern, with Type 1 diabetes mellitus (T1DM) showing a higher prevalence in males compared to females [1, 2]. Given that many individuals affected by T1D are at reproductive age [3], the incidence of T1DM is closely associated with a decline in male fertility [4, 5].

The etiology of diabetic‐related male infertility is highly complex and not yet fully understood. It has been postulated that hyperglycemia may disrupt spermatogenesis, impair the function of the hypothalamic‐pituitary‐gonadal (HPG) axis, or promote the excessive generation of reactive oxygen resulting in the imbalance between oxidants and antioxidant system [5]. Recently, gut dysbiosis has emerged as a contributing factor in T1DM‐related male infertility. Improved gut microbiota has been shown to enhance spermatogenesis and male fertility in T1DM patients [6].

Probiotics, as microbiota‐directed interventions, have demonstrated efficacy in ameliorating obesity‐related low sperm quality [7], regulating reproductive hormone levels, and optimizing metabolic health [8, 9, 10], including diabetes. In addition, probiotics have also been suggested as a potential strategy to manage T1DM, with reports indicating attenuation of hyperglycemia and modification of gut microbiota composition [11, 12, 13]. Among the various probiotic strains, Lactobacillus species are particularly prominent within the lactic acid bacteria group. Lactobacilli supplementation can modulate gut microbiota composition, diversity, and function to maintain the gut microbiota ecosystem in humans and mice, facilitating health problems [14]. Despite these promising findings, there is a lack of research investigating whether Lactobacillus probiotic interventions can serve as a therapeutic strategy for managing T1DM‐related male infertility.

Previous studies evaluating sperm quality in rat models have predominantly focused on parameters such as sperm motility and progression, often overlooking the importance of sperm trajectory. However, these parameters alone are insufficient for a comprehensive assessment of sperm function, particularly in rats, where a curved circular path in a counterclockwise direction and progressive swimming patterns characterize sperm movement [15]. Therefore, in this study, spermatozoa's swimming trajectory and progression are all included to provide a more thorough evaluation of sperm function [16].

Building on prior findings, this study aimed to address existing research gaps by evaluating the potential of *Lactobacillus rhamnosus* PB01 (DSM 14870) supplementation in mitigating metabolic and reproductive dysfunctions in a streptozotocin‐induced T1DM male rat model. Key outcomes measured included body weight, hormone levels, insulin resistance, sperm quality parameters, and oxidative stress markers, providing valuable insights into the probiotic's therapeutic potential for improving metabolism, weight management, and male infertility associated with T1DM.

## 2 Experimental Section

This study was conducted at the Animal Facility at Aalborg University (Aalborg, Denmark). All experimental procedures were carried out following the guidelines and under approval from the “Ministry of Food, Agriculture and Fisheries of Denmark Animal Experiments Inspectorate” (no. 2022‐15‐0201‐01111).

### 2.1 Experiment Design

**FIGURE 1:**
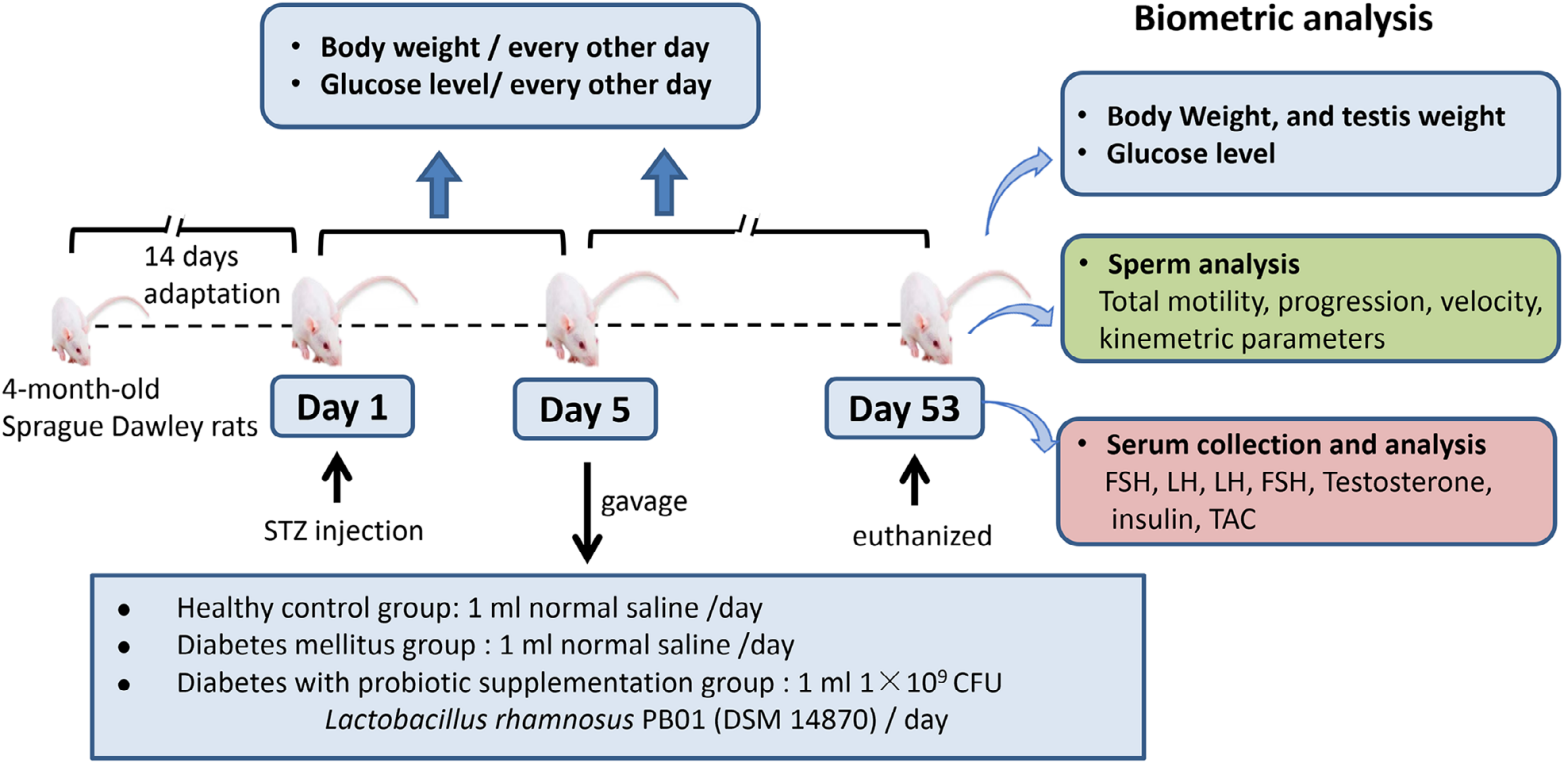
Graphical representation of the study's experimental design.

An overview of the study's experimental framework is illustrated in Figure 1. In brief, eighteen genetically outbred, 4‐month‐old Sprague–Dawley male rats (*Rattus norvegicus*; Janvier, France) were housed in ventilated cages (two rats per cage) using block randomization. The animals were kept in a quiet room at the temperature of 21 ± 1°C with 55 ± 15% relative humidity and 12‐h dark‐light cycles (light on from 08:00 to 20:00). Rats were allowed 14 days of acclimation period, and had ad libitum access to standard chow and tap water throughout the study.

After 14 days of acclimatization, 18 male rats were randomly allocated into nine cages (*n* = 2 per cage). Three cages (6 rats) were randomly selected as the healthy control (HC) group, and the remaining six cages (12 rats) were designated for diabetes induction. Diabetes mellitus was induced using the appropriate protocol. Five days post‐induction, the diabetic rats were further divided into a diabetes mellitus (DM) group (6 rats) and a diabetes with probiotic supplementation (DM+PB) group (6 rats).

The DM+PB group received a daily dose of 1 × 10^9^ CFU *L. rhamnosus* PB01 (ADM Denmark, Hundested, Denmark) suspended in 1 mL of normal saline. The DM and HC groups received 1 mL of PBS by gavage. Body weight and blood glucose levels were measured every other day for all groups throughout the experiment. At the end of the experiment, all rats were euthanized before semen, blood samples, and testes were collected for subsequent analyses.

### 2.2 Diabetes Induction

To establish the type I diabetes model after the acclimatization period, rats designated for diabetes induction received a single dose of 65 mg/kg body weight of streptozotocin (STZ; Sigma‐Aldrich, USA) dissolved in 5 mL phosphate‐buffered saline (PBS, Gibco, Taastrup, Denmark) subcutaneously.

To confirm diabetes induction, blood glucose levels were measured three days post‐STZ administration using the Contour Next glucometer (Bayer, Germany), with 50 µL blood samples collected via tail vein puncture. Successful induction of diabetes was determined by plasma glucose levels exceeding 18 mmol/L.

### 2.3 Probiotic Dosage Information

*L. rhamnosus* PB01 (DSM 14870; 1 × 10^11^ CFU) was provided as a lyophilized powder by ADM Denmark A/S (Hundested, Denmark). The lyophilized probiotic powder was aliquoted into test tubes and stored at −20°C until administration. Shortly before use, the prepared probiotic aliquots were serially diluted to 1 × 10^9^ CFU, using sterile normal saline as the diluent. The probiotic suspension was then stored at 4°C until administration.

For the experimental group (DM+PB), 1 mL of the probiotic suspension was administered orally via a gavage needle. The DM and HC groups received 1 mL of normal saline without probiotics in the same manner, to keep the negative effect of the mechanical stress of gavage. These procedures were conducted once daily throughout the study (Days 5–53).

### 2.4 Body Weight and Blood Glucose Level Examination

The body weight (±1 g) was measured every second day by placing the rats in a small bucket on a digital scale. Blood glucose levels were measured every second day, using the CONTOUR NEXT glucometer (Bayer, Germany) in a drop of blood (50 µL) collected from the tail. To prevent rats from presenting with blood glucose levels exceeding 20 mmol/L were administered 3 IU of NPH insulin (Novo Nordisk A/S, Denmark), an intermediate‐acting insulin, to prevent severe hyperglycemia and resulting morbidity and mortality. Blood glucose levels in rats receiving insulin were reassessed one hour after administration to ensure the efficacy of the intervention.

### 2.5 Euthanasia and Sample Collection

On Day 53, all rats were euthanized under general anesthesia with 2%–5% isoflurane and 95% oxygen administered via a rodent face mask connected to an anesthetic vaporizer (Harvard Apparatus, Massachusetts, USA). During anesthesia, the rat was placed on a heated mat (37°C) to prevent hypothermia. Following the induction of anesthesia, blood was carefully aspirated via cardiac puncture from the left ventricle prior to thoracotomy and excision of the heart. The testes and epididymides were collected by surgical dissection, and the testes were weighed (to the nearest 0.1 g). The collected blood samples were transferred into BD Vacutainer serum tubes (BD, Plymouth, PL6 7BP, UK) and allowed to clot undisturbed at room temperature for 20–30 min before being centrifuged at 1000–2000 × *g* for 10 min at 4°C to collect blood serum. The resulting serum was immediately aliquoted into 0.5 mL portions in Eppendorf tubes and stored at −80°C until further analysis.

### 2.6 Preparation of Sperm Suspensions

Sperm collection and preparation were conducted using a modified procedure previously described by Dardmeh et al. [7]. The cauda epididymis was carefully clipped from the testis and placed in 2 mL of pre‐warmed (37°C) Dulbecco's Modified Eagle Medium (DMEM) in 30‐mm Petri dishes. The epididymis was then cut open longitudinally to release the spermatozoa. The semen suspension was incubated for 30 min at 37°C to allow for complete dispersal of spermatozoa before proceeding to sperm motility analysis.

### 2.7 Sperm Analysis

Computer‐aided sperm analysis (CASA) evaluations were conducted using the Motility/Concentration module of the Sperm Class Analyzer (SCA; Microptic S.L., Barcelona, Spain). The SCA CASA system is comprised of a Nikon Eclipse 50i upright microscope (Nikon, Japan) coupled with a Basler Scout A780–54fc camera (Basler, Germany) and a 10x negative phase contrast objective (Nikon, Japan) in conjunction with a phase contrast condenser. For each rat, 25 µL samples were loaded into a CellVision 100 µm deep chamber slide (CellVision, Heerhugowaard, Netherlands), and one‐second videos (50 frames per second) were recorded from at least five fields.

The Motility/Concentration module of the SCA was utilized to categorize spermatozoa based on motility characteristics, including progression (progressive, non‐progressive, immotile), velocity (rapid, medium, slow, immotile), total motility (Tot. mot), and hyperactivated sperm (HA). Furthermore, kinematic velocity parameters, including straight‐line velocity (VSL), curvilinear velocity (VCL), and average path velocity (VAP), as well as motion path parameters, including linearity (LIN), straightness (STR), wobble (WOB), amplitude of lateral head displacement (ALH), and beat‐cross‐frequency (BCF) were evaluated as previously described by Dardmeh et al. [7].

### 2.8 Serum Analysis

Prior to analysis, serum samples were thawed overnight at 4°C. Serum levels of follicle‐stimulating hormone (FSH), luteinizing hormone (LH), testosterone, and insulin were quantified using commercially available ELISA assay kits (MyBioSource, San Diego, USA) according to the manufacturer's protocols. Total antioxidant capacity (TAC) was also assessed using a Colorimetric Assay Kit (MyBioSource, San Diego, USA) following the manufacturer's protocols. All analyses were performed in duplicate to ensure accuracy and reliability. The homeostasis model assessment index of insulin resistance (HOMA‐IR) was calculated as *fasting glucose (mMol*/*L) *×* fasting insulin (µU*/*mL)* / *22.5* [17].

### 2.9 Statistical Analysis

Data were analyzed for normal distribution using the Shapiro–Wilk test and presented as means ± standard deviation (SD) unless stated otherwise. One‐way Analysis of Variance (ANOVA) was used to compare sperm progression, velocity, hyperactivated sperm, kinematic velocity parameters, and motion path parameters among groups. Similarly, Glucose levels, serum hormone levels (LH, FSH, Testosterone, and insulin), TAC, and testes weight were analyzed using one‐way ANOVA to assess the differences among groups. A repeated‐measures analysis of variance (ANOVA) was used to compare the body weight among the groups and at different experimental days. The total number of motile sperm in different groups was analyzed using Kruskal–Wallis's independent sample test. Spearman's correlation analyzed the correlation among hormone levels, glucose, testes, and body weight. Statistical analyses were conducted using GraphPad Prism 10 (version 10.1.2, California, USA) and SPSS software (Version 29; IBM, Corporation, Armonk, NY, USA). *p* < 0.05 was considered statistically significant.

## 3 Results

Throughout the study, two rats from the diabetic mellitus (DM) group exhibited a substantial reduction in glucose levels approaching normoglycemic values. It showed a noticeable weight gain 2 weeks after diabetes induction. These rats were excluded from the data analysis to maintain the integrity of the diabetic model. Furthermore, another rat from the *L. rhamnosus‐*supplemented diabetes (DM+PB) group was excluded due to an extremely low total number of sperm in the semen suspension, chylous blood.

Consequently, the final number of rats included in the experiment comprised 4 in the DM group, 5 in the DM+PB group, and 6 in the healthy control (HC) group.

### 3.1 Blood Glucose Levels

**FIGURE 2:**
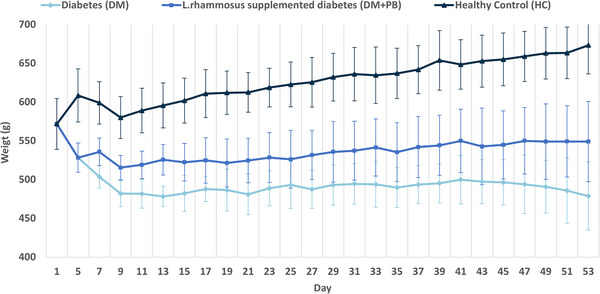
The mean (±SD) of glucose level in the healthy control (HC; *n* = 6), diabetes (DM; *n* = 4), and the *L. rhamnosus* supplemented diabetes (DM+PB; *n* = 5) groups throughout the study.

The glucose level changes of the rats across the experimental groups (HC, DM, and DM+PB) were systematically monitored throughout the study, as shown in Figure 2. Successful induction of diabetes, characterized by sustained hyperglycemia, was confirmed by the significantly elevated glucose levels in both the DM group and the DM+PB group compared to the HC group (*p* < 0.0001) on day 5. This significant difference in glucose levels was consistently observed throughout the study.

**FIGURE 3:**
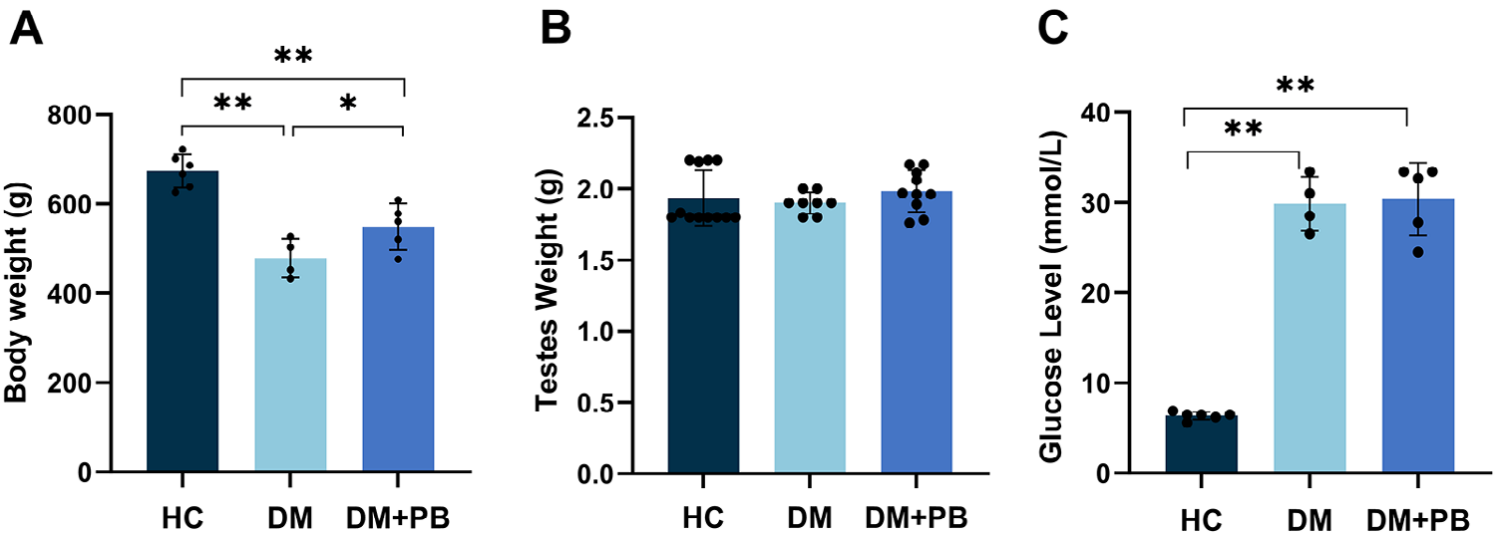
Estimated marginal means of (A) body weight, (B) testes weight, and (C) blood glucose levels at the end of the study (Day 53) across the healthy control group (HC; *n* = 6), Diabetes group (DM; *n* = 4), and the *L. rhamnosus* supplemented diabetes group (DM+PB; *n* = 5). Solid dots present individual data points, error bars present standard deviation. *p < 0.05; **p < 0.01.

The DM+PB group showed maintained comparable hyperglycemic states as DM group by the end of the study (*p* = 0.65) (Figure 3C).

### 3.2 Body Weight and Testes Weight

**FIGURE 4:**
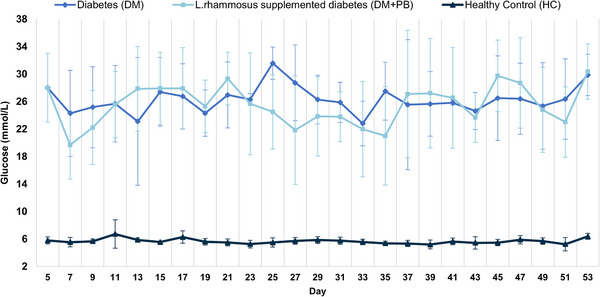
The mean (±SD) of body weight in the healthy control (HC; *n* = 6), diabetes (DM; *n* = 4), and the *L. rhamnosus* supplemented diabetes (DM+PB; *n* = 5) groups throughout the study.

The body weight of the rats across the experimental groups (HC, DM, and DM+PB) were systematically monitored throughout the study, as shown in Figure 4. The weight change could be divided into three conceptual phases: the diabetes induction phase (Days 1–5; initial 5 days following diabetes inducement), the gavage adaptation phase (Days 5–9; initial administration of either normal saline or probiotics on gavage), and the intervention and monitoring phase (Days 9–53; continuation of the gavaging with normal saline or probiotics).

By the end of the diabetes induction phase (Day 5), diabetic rats exhibited a significant reduction in body weight compared to the HC group (*p* = 0.001). This marked weight loss was maintained throughout the study.

During the gavage adaptation phase (Days 5–9), a pronounced and sudden decrease in body weight was observed in both the HC (*p* = 0.002), and DM (*p* = 0.038) groups from day 5 to day 9. However, probiotic supplementation in the DM+PB group appeared to mitigate the stress response associated with gavage administration, resulting in stable body weight (*p* = 0.204) that was significantly higher than that of the DM group on day 9(*p* = 0.038).

During the intervention and monitoring phase (Days 9–53), the HC group showed a progressive increase in body weight over time, with a significantly higher body weight observed on Day 19 compared to Day 9 (*p* = 0.03), continuing to elevate through to the end of the study (Day 53). On the other hand, the trend toward lower body weight in the DM group and higher body weight in the DM+PB group resulted in a significantly greater body weight in the DM+PB group compared to the DM group, beginning on Day 47 (p = 0.046) and continuing through to the end of the experiment on Day 53 (*p* = 0.035). By the end day, diabetes‐induced weight loss was mitigated by approximately 13%.

Furthermore, diabetic rats exhibited lower body weight and hyperglycemia than the HC group. A strong and statistically significant negative correlation was observed between glucose levels and body weight (*p* < 0.001, *r*_s_ = −0.792) (Figure 6C).

No statistically significant differences in testis weight were observed among the HC, DM, and DM+PB groups (Figure 3B). However, a significantly positive correlation was found between blood serum testosterone level and testes weight (*p* = 0.001, *r*_s_ = 0.564) (Figure 6F).

### 3.3 Hormone Levels and Antioxidant Capacity

**FIGURE 5:**
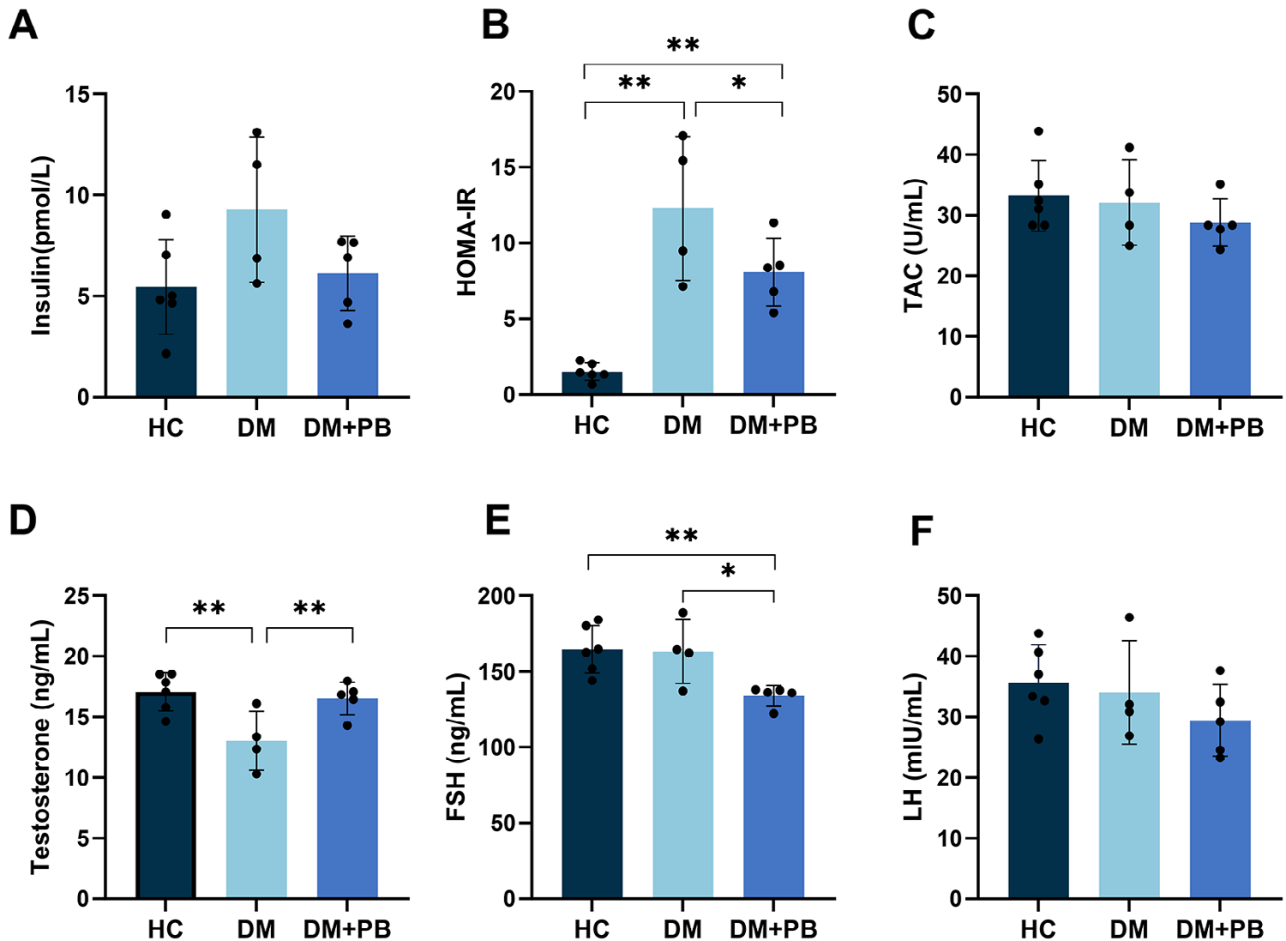
Mean (± standard deviation) blood serum levels of insulin, luteinizing hormone (LH), follicle stimulating hormone (FSH), testosterone, total antioxidant capacity (TAC), and homeostasis model assessment index of insulin resistance (HOMA‐IR) in the healthy control (HC), diabetes (DM), and *L. rhamnosus* supplemented diabetes (DM+PB) groups, at the end of the study. Solid dots present individual data points, error bars present standard deviation. *p < 0.05, **p < 0.01.

On the final day of the experiment, all three groups demonstrated similar insulin levels (Figure 5A). The DM group exhibited significantly higher HOMA‐IR values compared to the HC group (*p* < 0.0001) (Figure 5B). The DM+PB group showed significantly lower HOMA‐IR levels relative to the DM group (*p* = 0.012), although these levels remained significantly higher than those observed in the HC group (*p* < 0.001) (Figure 5B).

All reproductive hormone levels—luteinizing hormone (LH), follicle‐stimulating hormone (FSH), and testosterone showed a higher trend in the HC group compared to the DM and DM+PB groups (Figure 5D–F). In detail, the testosterone level was significantly lower in the DM group than in the other two groups. Testosterone levels in the DM+PB group were comparable to those of the HC group (*p* = 0.759), which were significantly higher than the DM group (*p* = 0.001) (Figure 5D). The DM+PB group showed a lower level of FSH (*p* = 0.003) compared to the DM group, while LH did not show a significant difference among the groups (Figure 5E,F).

**FIGURE 6:**
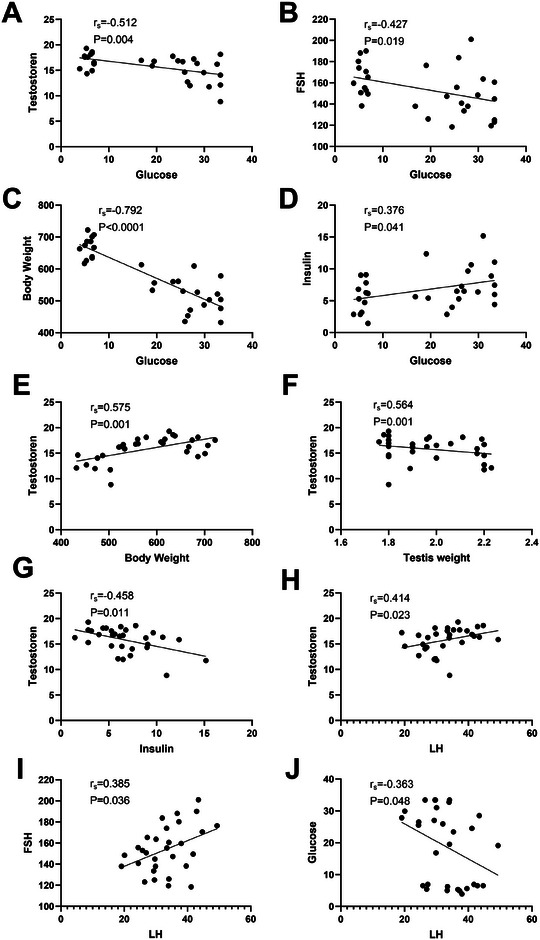
Significant associations (Spearman's correlation) among the reproductive hormone levels, insulin, glucose level, testes weight, and body weight, including data from rats in all healthy control (HC), diabetes (DM), and *L*. *rhamnosus* supplemented diabetes (DM+PB) groups (*n* = 15).

No significant differences in antioxidant capacity were observed among the three groups (Figure 5C). Spearman correlation analysis indicated that glucose levels were negatively correlated with the secretion of all assessed reproductive hormones, with higher glucose levels being associated with lower testosterone (*p* = 0.004, *r*_s_ = −0.512), FSH (*p* = 0.019, *r*_s_ = −0.427), and LH (*p* = 0.048, *r*_s_ = −0.363) secretion (Figure 6A,B,J). Specifically, in addition to glucose, insulin was also negatively correlated with testosterone secretion (*p* = 0.011, *r*_s_ = −0.458) (Figure 6G); in contrast, body weight was positively correlated with testosterone secretion (*p* = 0.001, *r*_s_ = 0.575) (Figure 6E). Additionally, there was a positive correlation between LH and testosterone secretion (*p* = 0.023, *r*_s_ = 0.414) (Figure 6H), while FSH did not correlate with testosterone. The positive correlation between LH and FSH was also observed, indicating that these levels tend to increase or decrease simultaneously (*p* = 0.036, *r*_s_ = 0.385) (Figure 6I).

### 3.4 Comparative Analysis of Sperm Motility and Kinematic Parameters

Sperm motility and detailed kinematic parameters are presented in Tables 1 and 2, respectively. The total percentage of motile sperm ranked from highest to lowest were in the HC group, DM+PB group, and DM group, although not statistically significant. Sperm motility progression (progressive, non‐progressive, immotile) and velocity (rapid velocity, medium velocity, and slow velocity) showed the same levels among groups (Table 1).

**TABLE 1:** Mean (± standard deviation) percentage of sperm in different progression and velocity categories, and total motile percentage in diabetes (DM), *L. rhamnosus* supplemented diabetes (DM+PB), and healthy control (HC) groups at the end of the study.

Group	Progression (%)			Velocity (%)				Total motile (%)
	IM	NP	PR	IM	Vel.S	Vel.M	Vel.R	
HC	30.28 ± 2.90	67.07 ± 2.85	2.65 ± 0.62	30.28 ± 2.90	31.12 ± 4.37	28.33 ± 2.61	10.26 ± 2.43	69.72 ± 2.90
DM	42.56 ± 14.81	53.92 ± 13.68	3.52 ± 1.30	42.56 ± 14.81	22.40 ± 9.40	26.49 ± 7.39	8.55 ± 2.21	57.44 ± 14.81
DM+PB	37.20 ± 3.98	59.73 ± 3.79	3.07 ± 0.70	37.20 ± 3.98	26.43 ± 3.14	23.90 ± 2.84	12.47 ± 3.43	62.8 0± 3.98

Abbreviations: IM, immotile; NP, non‐progressively motile; PR, progressively motile; Vel.M, medium velocity; Vel.R, rapid velocity; Vel.S, slow velocity.

**TABLE 2:** Mean (± standard deviation) values of sperm motility and kinematic parameters in diabetes (DM), *L. rhamnosus* supplemented diabetes (DM+PB), and healthy control (HC) groups at the end of the study.

Group	Sperm velocity parameters (µm/s)			Motion path parameters (%)				BCF (Hz)	Hyperactivated (%)
	VCL	VAP	VSL	STR	LIN	WOB	ALH		
HC	89.28 ± 4.42	52.37 ± 5.25^a^	15.95 ± 3.19^c^	29.71 ± 3.33^e^	17.44 ± 2.8^g^	58.29 ± 3.56^i^	3.18 ± 0.09	4.32 ± 0.47	2.22 ± 0.76
DM	92.12 ± 6.35	61.24 ± 4.09^a,b^	27.69 ± 3.61^c,d^	42.18 ± 2.71^e,f^	28.6 ± 2.65 ^g,h^	65.46 ± 2.58^i,j^	3.12 ± 0.11	4.87 ± 0.40	1.39 ± 0.29^k^
DM+PB	93.14 ± 4.80	52.32 ± 3.40^b^	16.7 ± 2.24^d^	30.76 ± 2.97^f^	17.22 ± 1.5^h^	55.8 ± 2.66^j^	3.25 ± 0.09	4.38 ± 0.32	3.51 ± 1.29^k^

*Note*: Similar letters within each column indicate pairwise significant differences among groups.

Abbreviations: ALH, amplitude of lateral head displacement; BCF, best cross frequency; LIN, Linearity index; STR, straightness; VAP, average path velocity; VCL, vurvilinear velocity; VSL, straight line velocity; WOB, Wobble.

Regarding sperm velocity parameters, the DM group exhibited significantly higher straight‐line velocity (VSL) values compared to both the HC and the DM+PB groups. However, the curvilinear velocity (VCL) did not show statistically significant differences among the three groups. With an accompanying trend, average path velocity (VAP) was higher in the DM group compared to the HC and DM+PB group. Sperm moving motion, including straightness (STR), wobble (WOB), and linearity (LIN), showed higher values in the DM group compared to the HC and DM+PB groups. DM+PB showed comparable levels to the HC group for all the above parameters.  Amplitude of lateral head displacement (ALH) and beat cross frequency (BCF) remained unchanged among the three groups. The DM+PB group exhibited significantly higher percentage of hyperactivated sperm than DM group, but comparable to HC group (Table 2).

## 4 Discussion

The potential of *L. rhamnosus* PB01 (DSM‐14870) to regulate weight and improve sperm quality in diet‐induced obesity mice models has been previously documented [7]. The present study examines the effectiveness of the same strain in the Type I diabetic rat model.

Type 1 diabetes (T1D) is an autoimmune destruction of islet β cells in the body leading to long‐term metabolic disorder, also developing hyperglycemia and varying degrees of insulin resistance [18, 19], which result of various complications, including weight loss, chronic inflammation, reproductive system dysfunction, and so forth. In this study, we observed the lower body weight in both diabetic groups (DM and DM+PB), and a correlation between significant weight loss and elevated glucose, which has been reported by previous findings in both diabetic patients and animal models [20, 21]. Notably, following 42 days of *L. rhamnosus* administration, DM+PB showed a higher body weight than DM and maintained until the end of the study. It endorsed again the protective weight management effect of the probiotic intervention, while this observation contrasts with previous findings in the high‐fat diet‐induced obesity mice model [7]. It should be noted that the weight change pattern in T1DM is different from that of the obese or general population, with initial weight loss in T1DM is associated with insulin deficiency, the body cannot effectively use glucose, leading to lipolysis and muscle catabolism metabolic consequences. The metabolic status‐dependent modulating effect of *L. rhamnosus* on body weight suggested that it might indirectly facilitate weight regulation by altering the gut microbiome function or generating key metabolic factors released into circulation that symbiotically with hosts to impart metabolite homeostasis and maintain systemic health weight management. Additionally, the initial weight loss following the gavage in both HC and DM groups could be attributed to the mechanical stress of the gavage procedure. We emphasize that this effect was not observed in the DM+PB group, suggesting that the *L. rhamnosus* intervention may mitigate the stress‐related impact on weight. This protective effect could be linked to the *L. rhamnosus* PB01 previously reported anti‐inflammatory properties and its nociceptive regulatory influence on mechanical sensitivity [22, 23].

Insulin resistance (IR) is a feature of type 1 diabetes [18, 19]. Homeostasis model assessment of insulin resistance (HOMA‐IR) is a clear indicator of insulin resistance [24]. The significantly higher HOMA‐IR values in both DM and DM+PB groups than in the HC confirmed the diabetes‐induced insulin resistance. Notably, the significantly lower values in DM+PB than in the DM group suggested that the *L. rhamnosus* supplement partially counteracted the metabolic dysregulation induced by diabetes. This beneficial effect may be attributed to its modulation of key signaling pathways involved in insulin sensitivity and systemic inflammation, as indicated by changes in TNF‐α, IL‐6, and IL‐10 levels, along with the upregulation of GLUT‐4 and Akt‐2 expression [25, 26, 27, 28, 29]. The regulation of PI3K/Akt/mTOR, AMPK/PI3K/Akt pathway signaling pathway may improve glucose utilization and consumption [29, 30], supporting their potential therapeutic role in diabetes management

While this significant improvement does not entirely reverse the insulin resistance as the IR in DM+PB is still higher than HC, further studies are required to assess whether administering the supplement for a more extended period, at a higher dose, or combined with additional therapeutic interventions could yield more pronounced effects.

IR and hyperglycemia negatively impact male reproductive function through multilayered and multifaceted mechanisms, including hypothalamic‐pituitary‐gonadal (HPG) axis dysfunction [31, 32], leading to abnormal LH, FSH, and testosterone secretion [27] and testicular dysfunction. A key aspect of HPG axis dysfunction is the reduced hypophyseal sensitivity to GnRH. This is supported by our findings, that the negative correlation between glucose and the secretion of reproductive hormones (LH, FSH, and testosterone), consistent with previous reports [33]. Low testosterone levels, also known as male hypogonadism [34], have been strongly associated with impaired sperm production [35, 36]. Lower testosterone level was observed in the DM group in the present study, similar to what previous research had observed in diabetic rat models [37, 38] and humans [27, 39]. This decreased testosterone levels could be due to diabetes‐induced reductions in Leydig cell numbers and functional impairments, evidenced by a loss of tyrosine phosphorylation and a marked decrease in the expression of GLUT‐3, androgen, and IGF‐I receptors [37, 40]. The slight reduction in LH levels was also observed in diabetic rats, which suggested that changes in Leydig cells may partially impact upstream LH targeting.

*L. rhamnosus* supplementation improves low testosterone status, with comparable testosterone levels observed in the DM+PB and HC groups, both of which were significantly higher than those in the DM group. Previous studies have demonstrated the efficacy of certain probiotic strains in modulating testosterone levels in humans [41, 42] and rats [43]; however, the effect of *L. rhamnosus* in this regard has not been previously reported. Recent studies have provided insights into the bidirectional relationship between gut microbiota composition and reproductive hormones, including testosterone [44, 45]. One study demonstrated that sex hormones, particularly testosterone, influence the host's gut microbiome composition and metabolite profiles in a sex‐specific manner [46]. In return, alterations in gut microbiota composition have been shown to affect testosterone levels, although the precise mechanisms remain under investigation [47]. Based on these findings, it is speculated that *L. rhamnosus* intervention may modulate testosterone levels by regulating the gut microbiome. Additionally, *L. rhamnosus* may increase testosterone secretion through direct or indirect modulation of the HPG axis and reproductive hormone levels. This hypothesis is supported by recent evidence suggesting that dietary supplementation with *Lactobacillus reuteri* can modulate hormonal regulation at the hypothalamic and pituitary levels [48], although further investigation is required to clarify the specific mechanisms involved. Another potential explanation is that *L. rhamnosus* may alleviate IR, thereby promoting the restoration of HPG axis function, enhancing testosterone secretion, and ultimately improving male reproductive potential, as previously observed by other strains of probiotic administration in human and animal studies in the literature [49, 50, 51, 52]. The positive correlation between testosterone levels and testis weight, consistent with previous studies [53]. Compared to the DM group, the significantly higher testosterone levels in the HC and DM+PB groups suggested that increased testosterone may contribute to the greater testis weight. Our results demonstrated that testes weight was highest in the DM+PB group, followed by the HC group, with the lowest observed in the DM group, although not significant. Testis size and weight serve as simple, noninvasive surrogate markers of spermatogenesis and reproductive health, which are commonly used as indicators of reproductive function [54]. Thus, it can be speculated that *L. rhamnosus* supplementation may potentially benefit addressing complex reproductive issues.

Diabetes is characterized by an imbalance between oxidative processes and antioxidant defense mechanisms, leading to a state of excessive oxidation, commonly referred to as oxidative stress. While physiological levels of ROS play a crucial role in maintaining cellular function and overall physiological homeostasis [55, 56], persistent hyperglycemia in diabetes contributes to excessive ROS generation, ultimately leading to tissue damage and playing a significant role in the pathogenesis of various diseases [57]. Sperm membranes, rich in polyunsaturated fatty acids (PUFAs), are particularly vulnerable to ROS‐induced lipid peroxidation, impairing motility and viability, potentially leading to diabetic male infertility [58]. Some probiotic strains have been reported to enhance antioxidant capacity, thereby exerting beneficial effects on the host [59, 60]. However, in our study, *L. rhamnosus* supplementation did not result in significant differences in antioxidant capacity among the different groups, suggesting that the observed improvements may not be primarily attributed to the mitigation of oxidative stress, thus we speculated that the improvements are more likely due to hormonal regulation and HPG axis modulation rather than oxidative stress alleviation as above discussed.

Sperm motility is regarded as the most vital indicator of sperm function. Although our study did not reveal statistically significant changes in total motility, a clear trend was observed: the HC group demonstrated the highest sperm motility, followed by the DM+PB group. In contrast, the DM group showed the lowest motility. The evaluation of progressive sperm motility, similar to previous studies [61], showed no significant differences among the groups. While it is important to note that rat sperm typically follows a circular motion path [15, 62] and progressive swimming movement, which was also confirmed by our visual inspection using the CASA system. In addition to categorizing sperm based on velocity, progression, and total motility, a more comprehensive evaluation of sperm kinematic details, including the swimming trajectory description, which are more sensitive markers of subtle changes in sperm motility [63], was carried out to gain deeper insights into subtle alterations in sperm function. We found that DM rats exhibited more linear or straight movement patterns, while the DM+PB rats showed levels comparable to those of the HC group. As previously mentioned, rat sperm typically follows a circular motion path; thus, these elevated linear parameters may be associated with poorer sperm quality [16]. Hyperactivation facilitates sperm penetration through thecumulus oophorus, the zona pellucida, and the oviductal mucus, which is regarded as having gained the ability to fertilize oocytes, is critical for sperm success of fertilization [64, 65]. The decreased hyperactivated sperm in the DM group implied an increased risk of fertilization disorders in this group. On the other hand, the DM+PB group had a pronounced positive effect on hyperactivated motility, further supporting the potential of probiotics in enhancing specific aspects of sperm motility and the overall fertilization capacity in diabetic conditions.

This study provides valuable insights into the potential benefits of *L. rhamnosus* PB01 (DSM 14870) in improving metabolic and reproductive health in T1DM rats. The findings suggest that *L. rhamnosus* PB01 may serve as a promising strategy for weight management and an adjuvant treatment to mitigate insulin resistance in T1DM. Moreover, it has the potential to partially mitigate diabetes‐induced reproductive dysfunction, possibly by alleviating insulin resistance and activating the HPG axis feedback loop to regulate hormonal levels, particularly testosterone. Further research with larger cohorts, investigating optimal supplementation periods, varied dosages, and by analysis of the gut microbiota composition, inflammation biomarkers and metabolomic analysis, to provide a more comprehensive underlying mechanisms and further highlight its clinical therapeutic potential.

## Supporting Information

Supporting information is available from the author upon request.

## Conflicts of Interest

H.A. is an external scientific consultant for Microptic S.L. (Barcelona, Spain). F.D. is an external scientific advisor for ADM Denmark A/S (Hundested, Denmark). These commercial affiliations did not alter our adherence to policies on sharing data and materials. ADM Denmark A/S (Hundested, Denmark) provided the commercially available probiotic supplements but did not have any role in the study design, data collection and analysis, decision to publish, or preparation of the manuscript.

## Data Availability

The data that support the findings of this study are available from the corresponding author upon reasonable request.
